# The complete genome of *Escherichia coli* JM101 assembled with a combination of Nanopore and Illumina platforms

**DOI:** 10.1128/MRA.00973-23

**Published:** 2024-01-16

**Authors:** Adelfo Escalante, Fernando Astudillo, Violeta Larios, Noemí Flores, Guillermo Gosset, Francisco Bolívar

**Affiliations:** 1 Departamento de Ingeniería Celular y Biocatálisis, Instituto de Biotecnología, Universidad Nacional Autónoma de México, Cuernavaca, Morelos, México; 2 Winter Genomics, Salavery, Ciudad de México, México; University of Notre Dame, USA

**Keywords:** *Escherichia coli* JM101, complete genome, F′ episome, Nanopore, Illumina

## Abstract

We report the complete genome and the plasmid (F′ episome) sequences of *Escherichia coli* JM101 assembled with a combination of Nanopore and Illumina data. The resulting genome is a single contig of 4,524,963 bp, and the plasmid consists of a single contig of 197,186 bp.

## ANNOUNCEMENT

*Escherichia coli* strain JM101 [*supE thi*Δ(*lac-proAB*)] carries the F′ episome [F′traD36 *proAB lacI*^q^Z ΔM15]. This strain was created to maintain the bacteriophage M13 for cloning vector purposes. The source was Joachim Messing, who described the strain (1–3). Strain JM101 was obtained directly from H. Boyer (UC San Francisco) and was part of Boyer’s laboratory’s strain collection, which was used for cloning the first synthetic genes of somatostatin and human insulin (4). In this contribution, we generate the complete sequence of the genome and the plasmid (F′ episome) of *E. coli* JM101 with a combination of sequencing data from Nanopore PromethION and Illumina NovaSeq PE150 platforms provided by Novogene Co., Ltd. (https://www.novogene.com/amea-en/).

An aliquot of a glycerol stock of the strain JM101 was streaked on an Luria-Bertani (LB) plate and overnight incubated at 37°C to recover single colonies. One colony was inoculated in LB broth at 37°C and 300 rpm, and genomic DNA extraction was performed with the DNeasy UltraClean Microbial Kit (QIAGEN, Hilden, Germany) according to the supplier’s directions. One-percent agarose gel electrophoresis was performed to evaluate the presence of a single band as chromosomal DNA integrity criteria. Ten micrograms of genomic DNA with A_260280_ = 1.8–2.0 ratio were sent to Novogene Co., Ltd. (Sacramento, CA), for library construction, quality control, and sequencing by Oxford Nanopore and NovaSeq platforms according to Novogene Co., Ltd., procedures. For library construction for the Nanopore platform, the high-molecular-weight DNA sample was optionally fragmented or size selected to obtain double-stranded DNA fragments with appropriate size. Then DNA fragments were end polished, nick repaired, and A tailed. The 1D library was produced by ligating sequencing adapters onto double-stranded DNA fragments. After purification with AMPure XP beads, the prepared library was ready for library quality control. For the NovaSeq platform, the genomic DNA was randomly sheared into short fragments. The obtained fragments were end repaired, A tailed, and further ligated with the Illumina adapter. The fragments with adapters were PCR amplified, size selected, and purified. The library was checked with Qubit and real-time PCR for quantification and bioanalyzer for size distribution detection.

Quality control for NovaSeq data (fq files) was performed with FastQC v0.12.0 (https://www.bioinformatics.babraham.ac.uk/projects/fastqc). Quality control for Nanopore reads (fasq files) was performed by the NanoPlot tool from NanoPack2 software (https://github.com/wdecoster/nanopack) (5). A FasQC analysis was also applied to all the fastq files from Nanopore sequencing. Sequence adapters and low-quality reads were removed with Trimmomatic v0.39 (6). NovaSeq sequencing generated 6,832,078 bp paired-end raw reads. After trimming, it resulted in 6,427,210 paired reads, whereas Nanopore sequencing raw data resulted in 85,386 reads with a median read length of 12,844 and a total of 1,235,170,827 bp.

We used the Flye assembler v2.9.2 (7) for genome assembling using only the Nanopore reads and polished with Pilon software v1.24 (8). The assembly quality was assessed by using the QUAST v5.2.0 (9). The assembling of Illumina NovaSeq and Nanopore sequencing data resulted in two contigs, one corresponding to the complete chromosome of 4,524,963 bp and the corresponding to the plasmid (F′ episome) of 197,186 bp. Gene annotation was performed by the NCBI Prokaryotic Genome Annotation Pipeline (10). Table 1 shows the genome and plasmid assembling and contigs annotation summary results.

**TABLE 1:** Features of the complete genome of *E. coli* JM101

Parameter	Genome	Plasmid (F′ episome)
Size (bp)	4,524,963	197,186
No. of contigs	1	1
G + C content (%)	50.77	49.88
No. of proteins^*a*^	3726	149
No. of rRNAs	22	0
No. of tRNAs	86	2
No. of N’s per 100 kbp	0	0
No. of N’s	0	0

^*a*^ According to PGAP, NCBI.

## Data Availability

The assembled genome sequences of the JM101 and the plasmid were deposited under BioProject PRJNA1000728, Biosample SAMN36776167, and SRA study SRP464031.
